# Diagnosis as Subculture: Subversions of Health and Medical Knowledges in the Orthorexia Recovery Community on Instagram

**DOI:** 10.1007/s11133-022-09518-2

**Published:** 2022-07-27

**Authors:** Amy A. Ross Arguedas

**Affiliations:** Reuters Institute for the Study of Journalism, University of Oxford, Oxfordshire, UK

**Keywords:** Medicalization, Healthism, Diagnosis, Subculture, Orthorexia, Recovery

## Abstract

Diagnoses are powerful tools that fulfill various practical and symbolic functions. In this paper, I examine how a contested diagnosis called orthorexia nervosa has been taken up by users on Instagram, where tens of thousands of posts engage with the topic, many of them from individuals who identify with the condition. I put scholarship on medicalization and diagnosis in conversation with literature on subcultures to foreground the subversive work that is enabled through this diagnosis. Drawing on more than 350 hours of online ethnographic fieldwork and 34 in-depth interviews, I examine how participants construct a shared identity, draw on common language and norms, and undertake collective practices, as they negotiate dominant understandings of health. I show how they draw on the legitimacy endowed by the diagnostic label to validate and make sense of experiences of suffering but also to counter dominant health-seeking discourses, practices, and aesthetics in an online space where these are highly visible and valued. I also discuss some ways Instagram as a digital platform shapes its uptake by this community in meaningful ways. On the one hand, participants draw heavily on the language and framing of medicine to make sense of their fraught experiences with food and their bodies, effectively advocating for the medicalization of their own suffering while also creating a sense of community and shared identity. However, on the other hand, they actively use the diagnosis and the recovery process enabled through it to effectively resignify dominant beliefs, values, and practices that are experienced as injurious, including some that are particularly prevalent on Instagram.

Diagnoses are remarkably powerful tools. As Jutel (2011a) has argued, diagnoses play a crucial role from a clinical perspective, explaining symptoms, defining prognoses, and determining prevention and treatment options. They fulfill important administrative functions, enabling access to insurance or sick leave (ibid). However, diagnoses are also important symbolically, legitimizing certain illness experiences and defining what we are prepared to accept as normal (Aronowitz 2008; Bryant 2011; Hutson 2011; Jutel 2009, 2011a). While some of these functions (i.e. administrative) remain tightly guarded by the medical profession, others (i.e., symbolic, and to a degree clinical) have become increasingly democratized, as medical knowledges permeate the general culture (McGann and Hutson 2011, xx-xxi). The present study sheds light on this trend and its implications by examining how one online community of mostly lay individuals on Instagram has taken up an unofficial diagnosis known as orthorexia nervosa.

The term “orthorexia nervosa” began to circulate in the late 1990s in the context of a growing obesity panic and corresponding anxieties about the nutritional intake of the population. This diagnostic label was proposed to describe a purported new eating disorder consisting of a pathological obsession with healthy eating, capable of disturbing emotional wellbeing and leading to malnutrition (Bratman and Knight 2000). Orthorexia is an unofficial diagnosis, not currently in the Diagnostic and Statistical Manual for Mental Disorders (DSM), although it has in recent years drawn growing interest from scholars and clinicians, and its inclusion in the DSM is under review by a special task force (Cena et al. 2019). However, even prior to this, it received considerable attention from the news media (Ross Arguedas 2020a, b) and had been adopted by a community of Instagram users who identify with the diagnosis and use the platform to address their recovery (Santarossa et al. 2019).

Examining the orthorexia recovery community on Instagram provides a rich entry point for understanding diagnosis as a social process and its relationship to medicalization. First, as an emerging and, to a degree, contested diagnosis, the uncertainty surrounding orthorexia demands explicit and ongoing work to assert its substance, boundaries, and legitimacy, as they cannot be taken for granted (Dumit 2006). Second, understanding how and why this diagnosis—and the knowledges coalescing around it—have been taken up and put to use in a non-clinical setting offers insight into the complex relationships between lay and medical knowledges—something that transcends the case of orthorexia, as the current pandemic has made clear (e.g., Au and Eyal 2022). And lastly, this case sheds light on the key role digital media can play in enabling and shaping medicalized identities and socialities while also demanding innovative research methods that are attentive to the particularities of such research sites.

The reliance on the popular social networking site Instagram for these purposes is of particular interest. A highly visual platform, Instagram is commonly thought of as detrimental to young women’s body image, a concern its parent company, Facebook (now Meta) has evidence to be true but has publicly downplayed, according to documents made public in the damning 2021 Facebook whistle-blowing account (Wells et al. 2021). The use of this platform for eating disorder *recovery,* while paradoxical in a sense, underscores precisely how important this platform is in mediating women’s perceptions of their own bodies.

In this paper, I draw on a digital ethnography and in-depth interviews to examine the work done through the orthorexia diagnosis in the Instagram community that has embraced it. I am particularly interested in how participants use orthorexia to negotiate the meanings of health and their relationship to it. To do so, I place scholarship on medicalization and healthism in conversation with concepts from subcultural studies to shed light on the shared identity, language, and practices that arise through the diagnosis, constituting a distinctive “group style,” and how these, in turn, are used to subvert, but also extend, medical logics. In addition, this study sheds light on some of the ways Instagram, as the digital technology enabling this community, shapes its uptake in meaningful ways.

## Review of the Literature

### The (Bio)Medicalization of Health Itself

Over the past several decades, scholars have developed a wealth of scholarship examining the increasingly central role of medicine in organizing social life. The concept of medicalization has been used to theorize the ongoing expansion of medical logics to more and more domains of human life (Conrad and Schneider 1980). The proliferation of novel diagnoses like orthorexia is illustrative of medicalization, as more behaviors and experiences are reinterpreted through the language of medicine. Initially, medicalization was conceptualized as a top-down process through which the medical profession expanded its jurisdiction and exercised control (Conrad 1992; Riessman 1983; Zola 1972). However, more recently, scholars have noted important shifts, as medicalization has intensified and become more complex, multi-directional, and multi-sited, what some have called an era of *bio*medicalization. (Clarke et al. 2003).

One defining feature of biomedicalization is a growing focus on health itself (see also Rose 2006), which offers a productive entry point for understanding why individuals may adopt increasingly extreme health-seeking behaviors, including dieting. In part, this tendency is shaped by a broader societal focus on risk (Beck 1992), which in the domain of medicine, has led to the blurring of boundaries between health and sickness, exempting nobody from risk-reducing practices (e.g., through diet and exercise) and health surveillance (Armstrong 1995; Fosket 2004; Rosenberg 1997). The emphasis on health has also involved a pursuit of bodily optimization as an ongoing project, one that favors an ideal of personal perfectibility and enhancement and maps onto neoliberal rationalities of consumer choice and self-improvement (Guthman 2009; Knorr Cetina 2005; Rose 2006).

The pursuit of health has become more than a possibility—it is an imperative that is implicated in a broader moral order (Lupton 1995). Crawford (1980) proposed the term “healthism” to refer to this expansive moralization of health, which is to be attained primarily through the modification of lifestyles at an individual level. In healthist societies, proper health—as an outcome and as a performance—becomes central to establishing moral worth, defining “good” and “bad” citizens (Lupton 1995; Polzer and Power 2016). As an ideology, healthism contributes to driving the contemporary fascination with diet and fitness, and the celebration of “health” as a reflection self-discipline and control (Brown 2015; Luna 2019; Smith Maguire 2008).

Second, biomedicalization is marked by transformations in the production and distribution of scientific knowledges, as biomedicine permeates the general culture (Clarke et al. 2003). Characteristic of this transformation is the blurring of experts and laypeople, a process further enabled through media technologies, as diverse actors participate in knowledge production and as sources of information multiply online (Hardey 1999; Miah and Rich 2008). It has also involved challenges to medical belief systems and practice, for instance, through the formation of embodied health movements, which focus on the lived experience of illness as a mobilizing force (Brown et al. 2004, 2011). Individuals, including those who identify with contested or unofficial diagnoses such as orthorexia, can seek knowledge or contribute to producing it online (Dumit 2006).

Third, biomedicalization has brought about a transformation of individual and social identities (Clarke et al. 2003). On the one hand, healthist mandates can be incorporated into a person’s sense of self, as they internalize, for example, being a “healthy” or “fit” person. On the other hand, people are more inclined to acquire and perform identities as patients and communities (Clarke et al. 2003, 183). Illness identities shape how individuals understand their experiences (Sulik 2011, 464). Collective illness identities are organized through shared narratives that unify dissimilar histories and give the illness experiences structure, meaning, and legitimacy (Barker 2002, 284). Again, new technologies like social media platforms have enabled the formation, extension, and transformation of support groups and diagnostic communities online, making medical conditions such as eating disorders increasingly public and shared (Conrad and Stults 2010; Conrad et al. 2016; Goldstein 2004).

### Diagnosis as Subculture

Following Jutel (2011b) and the disciplinary move towards a “sociology of diagnosis,” I approach diagnoses as a focal point of medicalization and as remarkably powerful tools, capable of symbolically framing experiences and defining normality. In the analysis that follows, I examine how the orthorexia diagnosis is taken up and strategically deployed on Instagram to achieve particular goals (Clarke et al. 2003, 183). To do so, I draw on scholarship from subcultural studies, which offers a helpful analytical foil for conceptualizing the identity, discourses, and practices that arise in the context of this community and how they serve to subvert medical logics. This is an analytical move more than a classificatory one: my goal is not to argue that the orthorexia community is a subculture rather than, say, a social movement or a support group. Instead, I borrow concepts from subcultural studies simply to illuminate meaningful dynamics that take place within the community, which in many ways resemble those of subcultures. I understand subcultures as “relatively diffuse social networks that have shared identities, distinctive meanings around certain ideas, practices, and objects, and a sense of marginalization from or resistance to a perceived ‘conventional’ society” (Haenfler 2013, 16).

There are striking parallels between illness identities such as orthorexia and subcultural ones, making the subcultural framework productive for the case at hand.^1^ First, illness identities often involve stigma. It is no coincidence that Goffman’s (1963) exposé about “spoiled identities” drew heavily on cases of mental illness. While medicalized approaches to deviant behaviors may partially remove blame from the individuals experiencing them, those diagnosed nonetheless maintain a status of “second-class” citizens (Conrad and Schneider 1980, 249). Similarly, subcultures are typically characterized by their condition of marginalization or “alterity,” which describes the distinct experience of “being alien or unrecognizable to the mainstream” (Lingel 2017). Second, collective illness identities, especially those emerging around contested diagnoses, sometimes cohere into an “oppositional consciousness” that seeks to undermine dominant ways of thinking (Brown et al. 2004; also see Mansbridge 2001). These practices resemble those adopted by subcultural groups “to resist the dominant culture by creating their own meanings” (Valentine et al. 1999, 13).

Beyond its attentiveness to experiences of marginalization and opposition, drawing on a subcultural framework foregrounds an understanding of how meanings in the orthorexia community are articulated through shared symbolic resources. Subcultural meanings and means of expression provide members with symbolic resources to negotiate the dominant meaning system in “their attempt to make sense of their own specific situation and construct a viable identity” (Murdock 1974, 213). Subcultures tend to develop a distinctive group style, which involves the “active organization of objects with activities and outlooks, which produce an organized group-identity in the form and shape of a coherent and distinctive way of ‘being-in-the-world’” (Clarke et al. 1993, 54). One common strategy adopted by subculturists is bricolage, which refers to “the re-ordering and re-contextualisation of objects to communicate fresh meanings, within a total system of significances” (Clarke 1993, 177). Through bricolage, subculturists appropriate objects and imbue them with new meaning, often in direct opposition to the broader, “dominant” culture. By adopting a subcultural lens, I am thus able to analyze how participants in the recovery subculture negotiate dominant understandings of health and their relationship to them precisely *through* their shared identity, language, and practices.

## Methods

This study is based on more than 350 hours of digital ethnographic fieldwork, or netnography (Kozinets 2010), and 34 virtual in-depth interviews with Instagram users who have regularly engaged in conversations about orthorexia, conducted mostly between January and August 2018. I selected Instagram as a fieldsite because it is home to a large community of individuals involved in eating disorder recovery, including a subset that identifies with orthorexia and uses the platform to support their recovery process (LaMarre and Rice 2017; Santarossa et al. 2019). Beyond the mere transmission of information, I understand the mediated communication of this community as enabling the construction of shared systems of meaning and a sense of commonness and communion, in line with a ritual view of communication (Carey 2002). Conducting ethnographic work online enabled me to understand these shared meanings and the practices through which they crystallized.

The digital ethnographic work I conducted was both enabled and constrained by the affordances of Instagram. One of the defining features of this platform is the lack of a clearly delimitated community or group to “enter” as an observer; rather, I had to construct the community myself, following individuals who were engaged in the conversations—and social networks—I was interested in understanding. To do so, during my fieldwork, I followed users who (a) had public accounts,^2^ (b) regularly engaged with orthorexia or related subject matter, and (c) did so in a way intended to connect with the broader conversation (via hashtags). As a proxy, I used one of two different criteria for determining what users to follow: recurrent use of orthorexia hashtags in posts or identification with orthorexia in their profile. I found users through searches of orthorexia hashtags (#orthorexia and #orthorexiarecovery) and through the social networks of those I was following, based on their own interactions. I also followed orthorexia hashtags themselves, which enabled me to organically encounter new accounts using those hashtags.^3^

Following IRB approval, I created a new Instagram account specifically for research purposes, where I posted two selfies and a brief description identifying me as a researcher currently studying orthorexia on Instagram. In my profile, I included a link to my personal website, where I described the research in further detail. Throughout my fieldwork, I followed over 150 accounts, some of them very active and others posting sporadically, and came across hundreds of others as I navigated the platform and hashtags. Upon following an account, I consistently liked the posts when I encountered them in my feed, in an ongoing effort to make my presence known. Some followed me back. Aside from regularly observing posts and stories, I limited my participation to liking.

My stints of fieldwork ranged between 15 minutes and six hours but typically lasted about two hours per day. I allowed my observation to flow across the platform, sometimes focusing on stories and other times focusing on posts. I also allowed myself to stray off the platform when users posted links or content from websites, blogs, and YouTube they wanted to call their followers’ attention to. This fieldwork was necessarily multimedial with an ongoing analysis of images, texts, videos, and other interactive features.

I also conducted online, in-depth interviews with 34 of the users I followed. I initially reached out to 68 participants via an Instagram direct message and/or email. I made an effort to contact individuals who represented the various categories of personal accounts I identified as predominant in the orthorexia space during fieldwork: individuals in recovery (most common), recovered individuals, coaches, and a handful of health professionals (see Table 1 for more information about the interviewees). In the message, I briefly described my research to them and inquired about their willingness to participate. Those who expressed interest (half) received a follow-up email with further details. Most interviews were conducted using a videoconferencing service; however, four responded via email due to personal anxiety, privacy issues, or limited connectivity. Prior to each interview, participants provided consent. The interviews allowed me to triangulate information from my ethnographic work regarding meaning-making and “behind the scenes” practices or experiences.

**Table 1:** Descriptive information about Instagram users interviewed

Pseudonym	Age	Gender	Account type	Followers	Race	Country	Interview duration	Interview date
Karen	35	Female	In recovery	316	White	USA	44 minutes	6/18/18
Olivia	24	Female	Recovered	2,621	White	USA	82 minutes	6/19/18
Erica	21	Female	In recovery	2,668	White	USA	60 minutes	6/19/18
Luna	29	Female	In recovery	336	White	Canada	78 minutes	6/25/18
Leah	34	Female	Coach	11,400	White	Canada	62 minutes	6/26/18
Marion	28	Female	In recovery	685	White	USA	63 minutes	6/27/18
Natalie	25	Female	Coach	994	White	USA	49 minutes	7/18/18
Emilia	32	Female	Coach	3,024	White	Estonia	52 minutes	7/22/18
Liam	18	Male	In recovery	249	White	USA	60 minutes	7/22/18
Eva	31	Female	Coach	370	White	USA	42 minutes	7/23/18
Agnes	21	Female	In recovery	550	White	Germany	61 minutes	7/29/18
Rachel	32	Female	In recovery	139	White	USA	60 minutes	7/30/18
Andrea	27	Female	In recovery	42	White	USA	85 minutes	7/31/18
Nora	28	Female	Coach	622	White	USA	68 minutes	8/2/18
Audrey	28	Female	In recovery	589	White	USA	59 minutes	8/2/18
Helen	34	Female	Coach	396	White	USA	73 minutes	8/3/18
Danielle	26	Female	Recovered	2,705	White	USA	67 minutes	8/4/18
Isabella	31	Female	Coach	31,000	White	USA	NA	8/7/18
Paul	47	Male	In recovery	119	White	UK	64 minutes	8/8/18
Ella	28	Female	Coach	92,000	White	Canada	53 minutes	8/9/18
Amber	31	Female	Recovered	316	White	USA	62 minutes	8/10/18
Jason	28	Male	In recovery	104	White	UK	49 minutes	8/11/18
Lana	19	Female	Recovered	1,064	White	Canada	63 minutes	8/12/18
Lauren	18	Female	In recovery	853	White	USA	69 minutes	8/16/18
Carmen	24	Female	In recovery	107	Latina	USA	39 minutes	8/20/18
Jenna	28	Female	In recovery	367	White	USA	81 minutes	8/25/18
Anne	21	Female	In recovery	197	White	USA	71 min	8/31/18
Clara	24	Female	In recovery	247	White	Netherlands	NA	8/31/18
Matilda	22	Female	In recovery	4,559	White	UK	NA	8/31/18
Charlotte	27	Female	In recovery	724	White	UK	46 minutes	9/3/18
Mariana	24	Female	In recovery	3,822	Latina	Mexico	NA	9/4/18
Johanna	36	Female	Professional	8,747	White	USA	41 minutes	9/5/18
Dana	29	Female	Professional	2,254	White	USA	43 minutes	9/6/18
Julia	29	Female	Professional	5,360	White	USA	52 minutes	9/6/18

Articles without interview duration were conducted in written form. Account sizes were recorded at the time of the interview

I analyzed the ethnographic fieldnotes and interview transcriptions with MAXQDA software using grounded theory, which involved simultaneous data collection and analysis. I inductively developed my understanding through ongoing and increasingly refined coding, data collection, and memo-writing to develop the conceptual categories (Charmaz 2001, 2006). In the analysis below, I explore how the recovery accounts cohered into a unique subculture of sorts, with: (a) a shared identity, (b) unique language and norms, and (c) common resignification practices, which in many ways countered widespread health imperatives and perfectionistic depictions of the self that proliferate on Instagram but did so by drawing on medical logics.

### Positionality and Ethics

My identity and positionality shaped my fieldwork. As a white Latina young woman, my identity in many ways overlapped with the participants of the community, which skewed young, female, and white. I was closely acquainted with Instagram as a user of the platform and was also familiar with eating disorder recovery communities online, which I participated in some time before beginning my fieldwork, as I recovered from my own eating disorder. I also previously occupied the role of an eating disorder patient in a formal treatment setting. While these prior experiences demanded ongoing reflexivity on my part as a researcher, they also gave me a much deeper understanding of the jargon, practices, and experiences there.

Doing online ethnographic fieldwork poses important ethical dilemmas (e.g., Urbanik and Roks 2020). The unwieldly nature of the community on Instagram as one constituted through webs of connections rather than a contained group meant that it was impossible to collectively ask for consent, and the volume of users I encountered daily made individual consent unsustainable for the ethnographic portion of the study. While I only examined posts by participants with public accounts, I understand that ordinary users are often unaware their data can be used by researchers (Fiesler and Proferes 2018). Within these constraints, I attempt to embody principles of a feminist ethics of care, anchored in an attentiveness to context, a desire to lift up voices that are at the margins, and efforts to do no harm (Luka et al. 2017). When possible, I draw on examples from participants I interviewed (who provided explicit consent) and those with the most public facing accounts. I have taken measures to protect the identities of the users, such as using pseudonyms, tweaking words from posts, and “intervening” all images (with filters and eliminating clearly identifying data) to make users and posts less identifiable and traceable (Hine 2015).

## Findings

### Recovery Identities and the Validation of Suffering

One irony of orthorexia as “an illness you have to fight to get” (Dumit 2006) is that in vying for the recognition of their distress online, orthorexia-identifying individuals must assume a stigmatized identity—that of being mentally ill. This is not unlike other contemporary subculturists who “in a sense ‘choose’ their marginalization” (Haenfler 2013, 17). Aware of this fact, many participants in the community, especially those who were actively in recovery (i.e. not yet recovered), tended to withhold information that would connect their recovery accounts with their offline identities, for instance, avoiding last names or images where their faces were highly visible. However, the diagnosis also provided an answer to the question of what was “wrong” with them in a context where they believed to be doing all the “right” things for their health, yet were feeling increasingly unwell, physically and emotionally. Many described their discovery of the diagnosis as epiphany-like moments. Erica,^4^ who was 21 years old, recalled how clearly the diagnosis resonated: “Once I read about orthorexia, I was like, ‘Oh my gosh, this was me.”’ Amber, 31, described the comfort she found in discovering the term: “That diagnosis, once you hear it, you’re like, ‘Oh my God, I’m not crazy, like, this is a real thing.’” Frequently, this discovery took place online.

However, characterizing this identity as an *illness* identity to an extent misrepresents the focus of identification: beyond identifying as orthorexic, they symbolically distanced themselves from the illness by adopting a *recovery* identity, a practice that has been noted in previous research about people recovering from addiction and eating disorders (Hastings et al. 2016; McNamara and Parsons 2016). This identification marked a stark distinction between the recovery community and the pro-eating disorder communities that also exist on Instagram. Thus, participation in the recovery subculture was contingent on framing experiences in terms of healing or in direct opposition to the illness; that is, opposing extreme health-seeking behaviors.

Recovery accounts often made this point in their usernames and profiles, clarifying their adherence to recovery with descriptions like: “vegan anorexia and orthorexia recovery” or “ED recovery warrior.” These accounts were typically situated vis-à-vis their recovery status: in recovery, recovered, or helping others recover. The recovery identity is a liminal identity, located somewhere between health and illness. It is also a striving identity based on an ethos of self-improvement, although the substance of the betterment project counters the fitness and weight-loss discourses that proliferate on Instagram and in public health promotion. Attaining recovery was not simply about returning to the same state prior to the illness but adopting a new, oppositional approach to healthism.

**Fig. 1:**
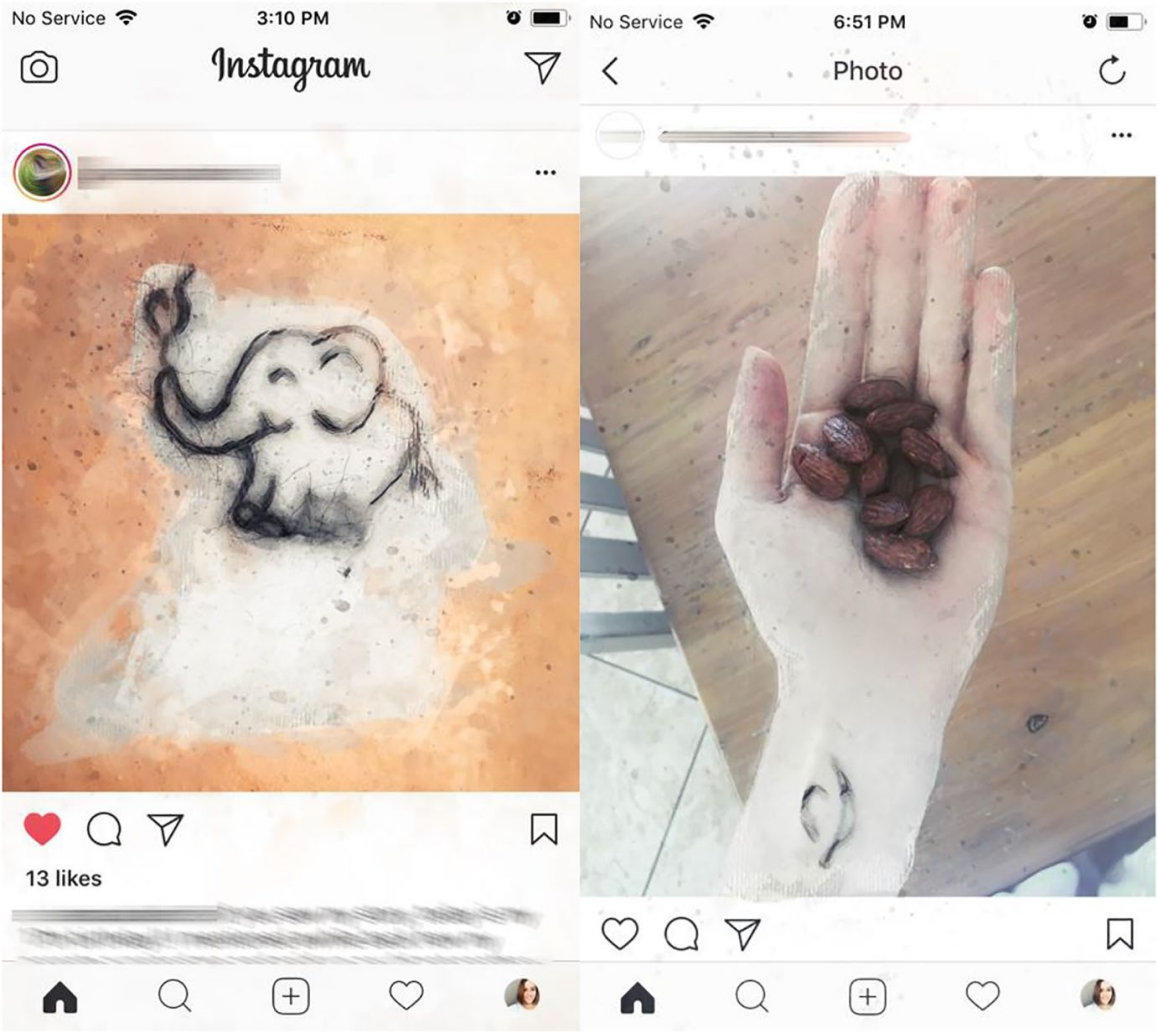
Photographic intervention of recovery tattoo posts

In addition to the shared use of hashtags, participants marked their membership in a variety of ways, for instance, posting pictures of themselves displaying a recovery symbol on clothing, accessories, or their bodies. The prevalent symbol was the National Eating Disorder Association logo, which resembles a flame or a heart. Multiple individuals shared pictures of tattoos with the symbol, which also served as a personal reminder to fight for recovery and a means of communicating their belonging to the community (Fig. 1).

Most individuals in orthorexia recovery had previously invested themselves in food-, health-, or fitness-oriented lifestyles and identities, such as veganism, clean eating, or body building, often with matching Instagram accounts. Thus, they transitioned from identities centered on health-seeking to illness identities focused on recovery—both illustrative of the identity transformations that characterize biomedicalization. Some deleted past accounts and replaced them with recovery ones, whereas other users slowly transitioned from one to the other on the same account, changing their descriptions and usernames in the process, and often deleting previous posts they now considered “disordered.” Some users tried negotiating a partial attachment to their past identities, especially when they could justify it ways that were compatible with recovery values (e.g., trying to recover while remaining vegan but for ethical rather than health-focused reasons), although there was widespread consensus that over-identifying with health and food was dangerous.

The changes that took place during recovery were often troubling as they left individuals questioning who they were. As one young woman reflected on a post: “I’m afraid of letting go of that ‘healthy persona’ I’ve lived my entire life” (Fieldnotes, 03/17/18). They also led individuals to experience a sense of marginalization or exclusion, either from their previous health and fitness communities or from the broader health-oriented culture. Charlotte, a 27-year-old in the UK, who had become very focused on fitness and clean eating, recalled the social validation she used to receive, both on and offline. “I had people coming up to me at the gym and people commenting, saying, ‘Oh, you look amazing,’ ‘You’re so dedicated,’ ‘How’d you get so lean?’ …People wanted to be like me,” she recalled. “Whereas, obviously, now I don’t get anything like that.” The status accrued through her past health-focused identity was difficult to let go of. However, through the diagnosis, she was able to reframe her health-obsessive—albeit status-building—past as pathological rather than ideal, while signifying her changes as healing.

Recovery identities also provided new pathways to acquire status. In the recovery community on Instagram, this status was signified, in large part, through Instagram’s metrics in the form of likes, comments, and follower counts. Some of the people I followed had come to acquire thousands or even tens of thousands of followers. Most were aware of these numbers and many discussed strategies to grow their followings. As Anne, a 21-year-old in recovery, acknowledged, it is “intimidating when you follow accounts that have like the 15K million followers, and you’re like, ‘Oh, I have 200.’ And Instagram is all about getting those followers.” Thus, while the recovery subculture foregrounded very different ways of relating to food and eating, it also inscribed Instagram’s logics in other ways.

Validation was central to this community. For instance, they validated that the orthorexic experience was real, dangerous, and deserving of help in a way that was not contingent on weight (like anorexia). As one young woman wrote in a post, “There is no ‘sick enough.’ Any eating disorder can kill. All eating disorders are dangerous and every sufferer, no matter their weight, needs help” (Fieldnotes 02/22/2018). Similarly, they validated decisions to change, something participants were often unable to find elsewhere. As Charlotte’s experience, above, shows, people outside of this context did not always understand what was “wrong” with their past behaviors, read as disciplined and healthy. Yet, since becoming a part of the community, “I do get a lot of comments from other people in recovery saying, ‘Well done, you look so much better…You look so much happier,’” Charlotte explained. Thus, in validating the realness of each other’s experiences and their decisions to change, they were also recognizing previous health-focused behaviors as disordered.

Yet validation—like status—was contingent on a person’s ability to make themselves seen by others in recovery, which given Instagram’s architecture centered on personal networks, largely depended on their ability to amass followers. This required a degree of skill and knowledge about the platform’s technical features and an awareness of the social norms and practices that were rewarded on it. I observed many instances of people, especially those new to the community or with few followers and weak connections, failing to receive support on posts where they expressed distress. In other words, support, as a resource, was unequally distributed on Instagram, concentrating more heavily among accounts that were most popular.^5^

### Creating a Group Style Through Shared Language and Norms

Like those in other subcultures, individuals in recovery often employed distinctive ways of communicating with each other. Central to this project was the uptake of Instagram “captions” for personal reflections and thoughts, which more than operating as descriptions of the pictures posted, were used as personal diaries to reflect on their experiences. Through written and multimedia texts, participants developed shared ways of communicating, which, in conjunction, contributed to the creation of a distinctive group style.

#### Therapeutic Language

Therapeutic language commonly used in eating disorder treatment was prevalent in the recovery space. Through their interactions, those who had been in therapeutic settings transmitted it to those who had not. Most obviously, participants used diagnostic labels frequently in their captions and hashtags, alongside terms like “restriction,” (self-imposed limiting or withholding of food for weight or health manipulation purposes) “binging,” (consumption of excessive amounts of food), and “purging” (efforts to eliminate or reduce the impact of perceived unhealthy foods through vomiting, laxatives, or exercise) to simultaneously describe and problematize the behaviors they were referring to. These behaviors were usually spoken about in the abstract.

For example, in a video story, one participant admitted to her followers that she was struggling with “really wanting to restrict again” (Fieldnotes, 01/15/18). Without requiring any further elaboration, this terminology both communicated to others that she was struggling with a compulsion to cut back her food consumption while framing the act as disordered, rather than, for instance, disciplined or controlled. Similarly, in another post, one user shared an experience she was proud of, where she had been able to eat and enjoy strawberries with sprinkled sugar on top, something her ﻿“orthorexic mind also would’ve SCREAMED at me” for doing in the past. In response, a friend she regularly interacted with reaffirmed her decision: “﻿I am so proud of you for choosing nourishment over restriction” (Fieldnotes, 01/27/18). This response drew on the shared language of “restriction” to frame the alternative option of withholding sugar as a form of restriction, which was tacitly reaffirmed as disordered and problematic.

Other therapeutic terms came up regularly. The notion of “using behaviors” involved a more abstract and preferable way of referring to eating disordered behaviors such as those mentioned above (e.g., “went a year without behaviors and then struggled again for a bit. It’s ok!! It doesn’t negate all that progress” [Fieldnotes, 06/11/18]). “Urges” referred to impulses to engage in disordered behaviors (e.g., “the guilt and urges are high, but I’m going to fight those urges and get on with my day” ([Fieldnotes, 02/21/18]). “Compensation” described inappropriate attempts to make up for or revert the impacts of having eaten certain foods (e.g., “[the] urges to compensate are high” [Fieldnotes, 08/06/18]). “Slips” or “slip ups” described anomalous lapses in an otherwise consolidated recovery process (e.g., “One slip does not mean you tumble all the way back down. It’s not too late to catch yourself” [Fieldnotes, 05/25/18]). Unlike “relapses,” which signal patterned and recurring usage of disordered behaviors, “slips” were considered exceptional and thus not compromising recovery. “Triggers” were stimuli that were believed to set off or elicit disordered behaviors.

Posts by individuals struggling with their recovery often included “trigger warnings” or more frequently “TW” (or PTW for a possible TW) to caution that content could be experienced as triggering by others. This content usually involved some degree of explicitness in discussing disordered behaviors or number talk involving weight and nutrition (pounds, calories, etc.). Users unfamiliar with these norms had to learn what was deemed triggering in the recovery context. Several newcomers I observed initially included calorie counts for all the meals they posted. However, they usually ceased doing this after realizing it was frowned upon—or on occasion, after getting called out. I documented one such instance in response to a post about weight gain by a user apparently unfamiliar with the norms, which contained exact weight details. Another user brought this to her attention:

Can I very gently say it can be difficult for people in recovery to see numbers? Mentioning the total pounds you’ve gained is not usually something we encourage in recovery circles and I’m sure with many of your followers being in recovery from an ED it may just be something worth taking into account. I love your message and page though! (Fieldnotes, 02/09/18)

Following a cordial back and forth, the original poster removed the numbers, and the only traces left were in the comments prompting their removal. It was not just that sharing numbers was considered upsetting to others but that knowing and documenting numbers, an expression of surveillance and control of food intake, was seen as conflicting with recovery and healing.

#### Diet Culture

“Diet culture” was a pejorative term, widely used in the recovery space to refer to the dominant, healthist culture: a wide assemblage of discourses and practices promoting dieting and weight loss, which permeate media, health promotion, and industries selling wellness and fitness products and services. Diet culture was viewed as oppressive, injurious, and deceitful, contributing to body dissatisfaction and eating disorders. It was also a politically loaded term, directly alluding to the broader culture that was believed to be at fault for orthorexia. Some identified as “anti diet culture” or “diet culture dropouts” in their bios, and many wrote about it or invoked the term in posts, for instance, suggesting their body hatred was “something that diet culture taught me” (Fieldnotes, 03/18/18).

Diet culture critiques often echoed academic commentary of healthism, underscoring the powerful but problematic entanglements of health and morality, and its ability to act upon the self. One dietitian described the concept to her followers, asserting that diet culture “tells us that our weight and the shape of our bodies is more important than overall health” and “tells you to not eat X, Y, Z because its ‘bad’; assigning morality to foods” (Fieldnotes, 03/24/18). Indeed, many criticized the reductionistic labeling of foods, encouraging others to stop categorizing foods as “good” and “bad.” Commentary against diet culture also tended to challenge practices designed to externally surveil or control the body, such as weighing and logging calorie intake, instead encouraging others to achieve freedom by trusting their own bodies. Erica, who had a recovery account and blog, shared a post where she wrote about her exasperation with people adhering to these “health obsessed behaviors’’:

It makes me want to stop and scream in their faces: “PLEASE DON’T DO THIS!! YOU ARE MISSING OUT ON LIFE!!! YOU DON’T HAVE TO BE A SLAVE TO THE NUMBER ON YOUR SCALE OR THE ‘QUALITY’ OF YOUR FOOD.” (Fieldnotes, 02/12/18)

Examining her own experience, she confessed that her anger derived from the fact that a part of her still wanted to participate, and how, despite having gone down that road and having “crashed and burned BIG TIME,” there was still “a little voice taunting” her, telling her it would work next time. Recovery is “so damn hard… when the world that surrounds us reinforces those voices by repeating the exact same things.” Thus, diet culture was described as working on the self through external pressure to conform but also through internalization, becoming “a little voice”—the orthorexia—that made recovery so difficult.

### Recovery Bricolage: Re-signifying Foods and Bodies

In addition to their shared language and norms, participants in the orthorexia community adopted a variety of resignification practices through which they transformed the meanings of quotidian “objects” or signifiers, typically involving food and bodies. I analyze two common examples of this “bricolage” to illustrate how it was deployed in this community.

#### Fear Food Challenges

Prior to recovery, individuals with orthorexia tended to have ample lists of foods that were off limits. These “fear foods” were typically items popularly read as unhealthy, such as those high in saturated fats and refined carbohydrates, whereas “safe foods” were more often vegetables and protein. As the term suggests, consuming even small amounts of fear foods was extremely distressing for many and often evoked overbearing feelings of shame, guilt, and even disgust. For those in recovery, these intense responses to food were read as indicative of the underlying pathology and thus needed to be overcome through practice, as explained by one recovery coach: “﻿The more you expose yourself to those foods the quicker you will come to normalize them, and they will lose their power over you” (Fieldnotes, 08/31/18).

To overcome fear foods, participants individually or collectively took on “fear food challenges” in which they would force themselves to consume distressing food items. Amber, a recovered coach, explained the challenges associated with the process: “You’re going to go through digestive issues and anxiety and fears because you’re introducing all these…fear foods. The goal is to get to a point where food is just food to you, where nothing feels off-limits anymore.” Danielle, a former athlete, who used her account intensively during her recovery from orthorexia, explained she would post pictures of her fear foods and reflect on the experience in the captions: “I really used to kind of dig down and go through all of my thoughts about a specific fear food or just anything that I was eating, everything that went around it.”

**Fig. 2:**
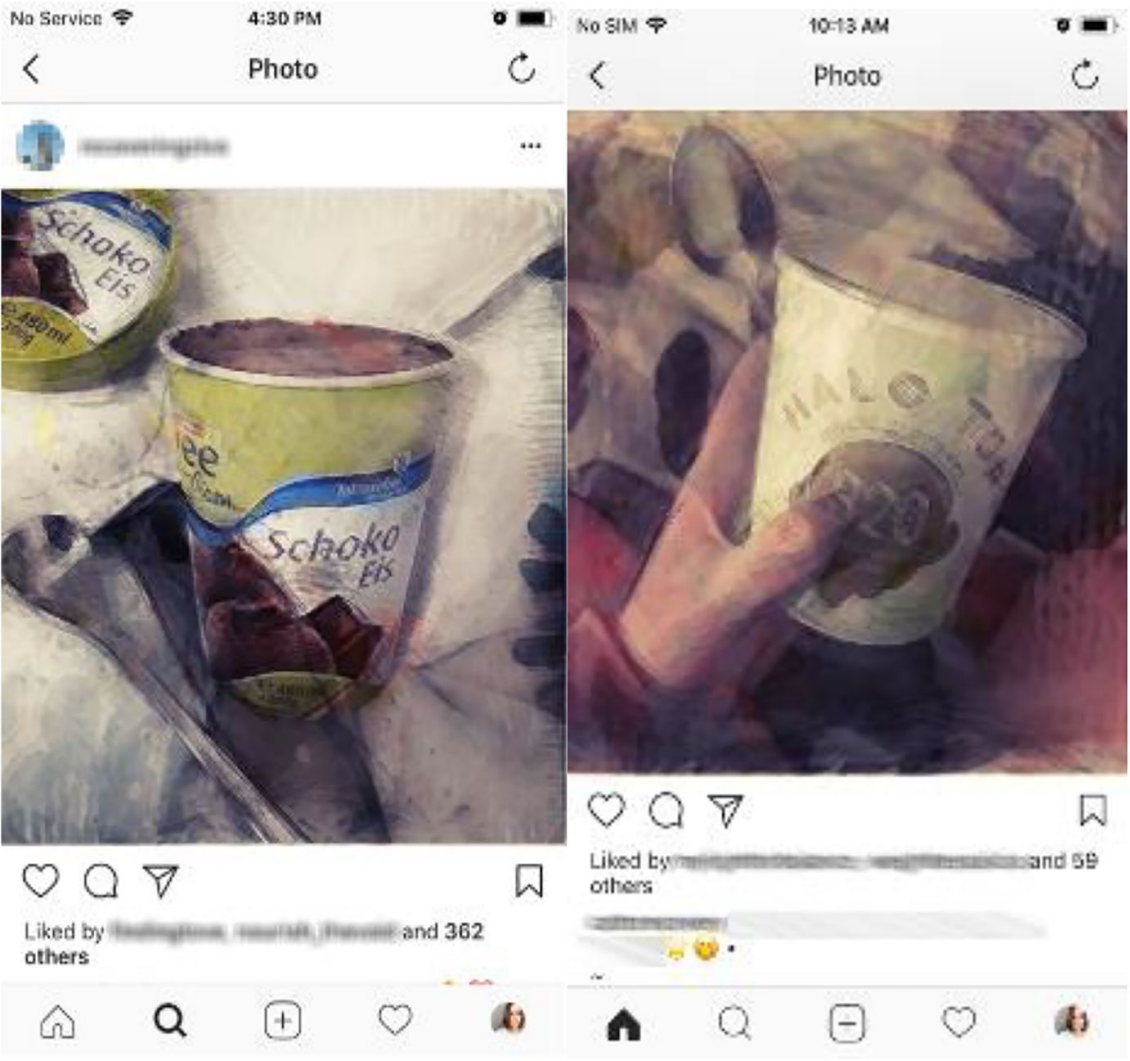
Photographic intervention of a “pint party” posts

Sometimes, fear food challenges involved group dynamics like #FearFoodFridays, in which participants undertook the challenge collectively and chimed into a stream of associated posts using the hashtag. One ritualized fear food challenge were “pint parties,” in which participants trying to overcome their fear of sweets would attempt eating a pint of ice cream and share a picture with a caption about the experience (see Fig. 2). Ben & Jerry’s, notorious for its high fat and sugar content, was particularly popular, although users also participated with other kinds of ice cream. One woman reflected on her pint party in a caption, noting, “Food freedom really changes your life. I’m enjoying ice cream just because tonight. I don’t need a reason to eat dessert anymore” (Fieldnotes, 08/28/18). She challenged others to undertake “something you’re craving that isn’t among your ‘safe’ foods.” Following a separate pint party, another young woman celebrated, writing, “#PINTPARTY COMPLETE! It was amazing and i feel great (also a bit sick)” (Fieldnotes, 06/01/18). By imbuing foods previously experienced as unhealthy with a therapeutic purpose, participants effectively re-signified these foods as healing. This restoration was experienced as liberating in that it allowed them to separate themselves from previously held food rules.

This is not how individuals in recovery ate most of the time. To the contrary, most accounts were flooded with pictures of “safe foods” such as salads, zucchini oatmeal, nut butters, etc. The meaningful meals for the sake of recovery, however, were those that challenged orthorexia—that is, they were “unhealthy” from a normative point of view. Fear food challenges must also be understood in the broader context of Instagram as a digital platform where health- and fitness-oriented dieting proliferates. Hashtags like #cleaneating and #keto amass tens of millions of posts, where sugary, high-fat ice cream is out of the question. Participants were keenly aware of this and used their fear food challenges to talk back to the idea that these highly pleasurable foods were intrinsically unhealthy, arguing instead that they were a crucial part of a truly healthy diet, one anchored in freedom rather than obsession.

#### Transformation Photos

A second resignification practice involved the reinterpretation of physical bodies, and perhaps more importantly, of certain kinds of physical change. Before-and-after pictures of bodies have long pervaded advertising for diet and fitness products, and more recently, have become popular on personal Instagram accounts of individuals documenting their own physical transformations.^6^ These photos are widely interpreted to communicate improvement; the “now” reflects the improvement of the “then.” In the context of prevalent weight-loss and fitness discourses and industries, the before version typically depicts more fat and less muscle, whereas the after photo shows a loss of fat and/or increase in muscle. However, in the recovery subculture, the assumption of weight-loss as superior was flipped on its head; the reappropriation of transformation photos often celebrated the increase in fat and the decrease in muscle during recovery, which was re-signified as healing.

The practice of sharing before-and-after photos is widespread among the anorexia recovery community, where visibly emaciated bodies are compared with their improved, recovered version—with more fat. To an extent, these bodies have been restored from their extreme thinness to a state of “normality.” However, orthorexia-identifying individuals were not always under-weight to start out, and their transformations often countered mainstream images more starkly. In fact, the “before” versions were often of very muscular or “fit” bodies that could easily have been the “after” version of fitness transformation images. And that was precisely the point of these posts: to bring into to question the widespread celebration of “ripped” bodies, to instead argue that the work it takes many people to produce them can be damaging.

Aside from using counter-normative images in the before and after slots, the resignification work was achieved through overlaid text in the image or captions that provided context for the “truth” behind the pictures. One example of this was a post shared by Ella, a recovery coach with a popular motivational account amassing tens of thousands of followers. The before and after image contained, on the left, a picture of herself some years earlier, holding up her shirt to show her six-pack abs, and on the right, a more recent picture where she is sitting in her underwear with thicker legs curled up and showing tummy rolls where the six-pack used to be. Ella began the post caption acknowledging:

This is not the before and after image you are used to seeing in the media. This is the before and after picture of a person who suffered in silence for over a decade, going across the entire spectrum and variety of eating disorders. (Fieldnotes, 03/18/18)

**Fig. 3:**
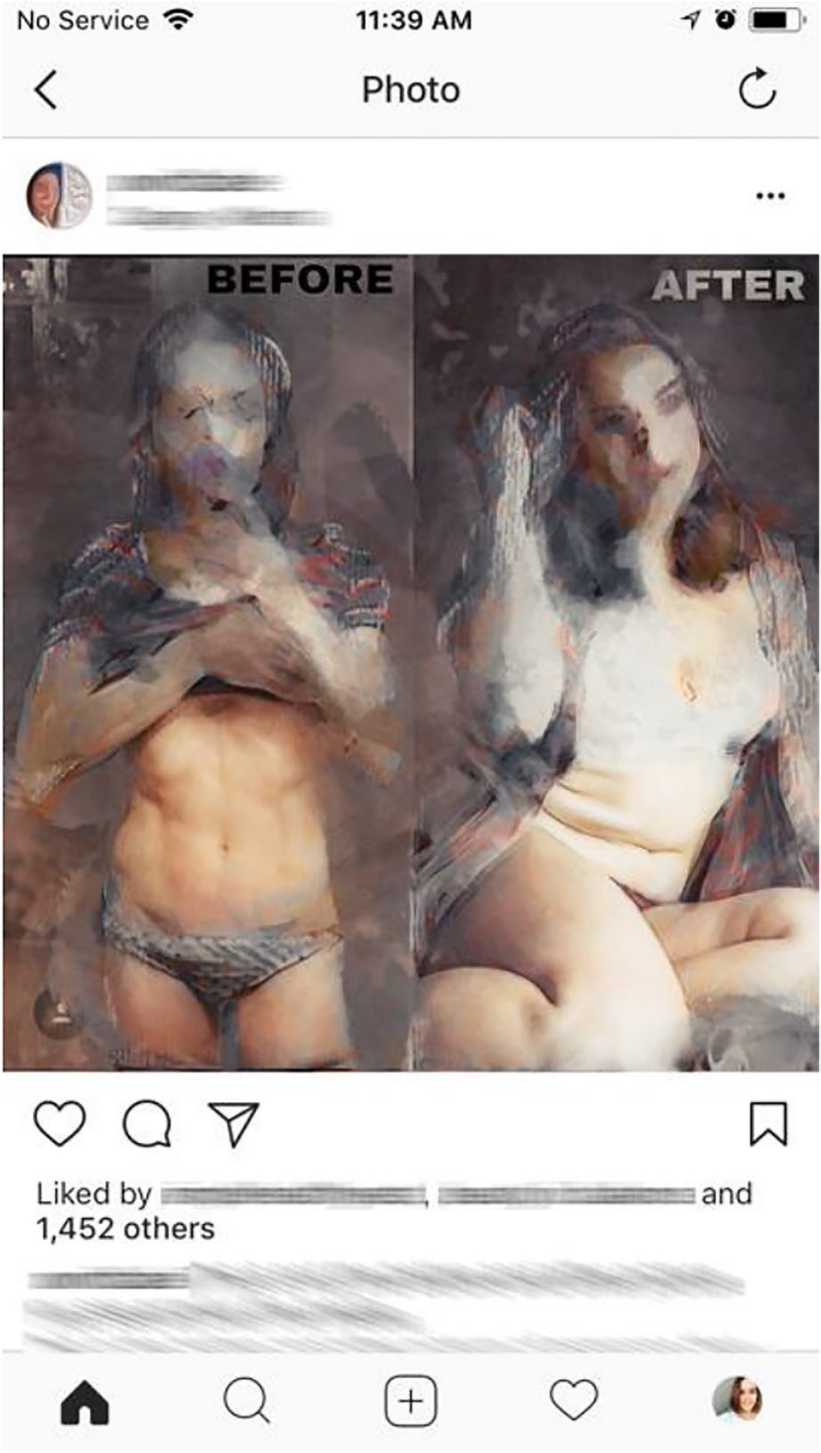
Photographic intervention of a transformation post

In the post, she described her experiences restricting, binging, purging, and over-exercising, and went on to list some of the rigid diets she had followed and her experiences body building. “On the outside, I looked like a healthy happy girl who was thin and fit. On the inside, I was ripping myself to pieces,” she revealed. The post urged caution in judging the weight gain of others, as they too may be suffering in trying to maintain lean or fit bodies. She added, “I would be lying if I claimed to love the after picture. But I recognize that hating the after body is something I learned from diet culture.” The post had hundreds of comments thanking her and praising her for sharing her struggles (Fig. 3).

Ella used the side-by-side format frequently not only to encourage her followers to celebrate non-normative changes in body size but also to criticize the filtered or highly produced character of Instagram photos more generally. For instance, she would compare two photos of herself standing versus sitting (and thus without versus with tummy rolls or cellulite), sucking her belly in versus relaxing it (and thus flat versus round), which she referred to as “Instagram versus reality” posts and were particularly popular. In doing so, she and others posting similar kinds of images were attempting to subvert ideals of what health looks like while also disrupting the before-and-after format in itself and its assumptions of what is better and worse.

Yet, Ella’s posts were not counter-hegemonic in every respect. While her recovered body was larger than it previously had been, it also did not stray from the norm to an extreme degree. In some respects, she coincided with more normative beauty standards, including a traditional presentation of femininity (e.g., hair, makeup, lingerie, etc.), not to mention her position as a young white woman. Furthermore, her photos were evidently produced with great care, often foregrounding her physical body, in line with the mainstream Instagram aesthetic, all of which contributed to the success of her account—which she also used to provide recovery support services, some of them at a cost.

## Discussion

This paper has examined how participants in the orthorexia recovery community on Instagram collectively use the diagnosis to negotiate the meaning of health and their relationship to it. I have drawn on a subcultural framework to illuminate how the diagnosis serves to create a shared sense of identity centered on recovery and restoration, which is enacted and developed through shared language, norms, and practices—a “group style” of sorts. Similar to other subcultures, these shared meanings and means of expression provide members with symbolic resources to negotiate dominant understandings of health while making sense of their own experiences and constructing viable identities (Murdock 1974). If we understand Instagram—the architecture, affordances, shared practices—as a site where the performance of idealized versions of reality is widespread and quotidian (including the proliferating diet, wellness, and fitness cultures), its consolidation as a crucial place for the subversive work of the recovery subculture is no longer a bizarre paradox; instead, it emerges as an obvious site for the contestation of meanings of health.

These meanings involve a complex relationship with the medical establishment and with medical logics more broadly. On the one hand, participants drew heavily on the language and framing of medicine to make sense of fraught experiences with food and their bodies, effectively medicalizing their own suffering while also creating a sense of community and shared identity. The use of a diagnosis—albeit unofficial—alongside a wide repertoire of medical language endowed these experiences with legitimacy, providing a means to acknowledge the distress and feelings of being out of control that had previously characterized their lives. This is not unlike other online forums and communities addressing contested illnesses, where participants reaffirm the medicalization of their own conditions, drawing on their lived experience (Barker 2008; Dumit 2006; Goldstein 2004, 128). This exemplifies several key features of biomedicalization, including the transformation of individual and social identities through diagnostic labels and changes to the production and distribution of scientific knowledges, as non-experts actively participate in addressing medical issues, largely based on their lived experience, but also drawing on the medical language that has increasingly permeated the culture and their own experiences.

On the other hand, participants actively used the diagnosis—and perhaps, more significantly, the recovery process enabled through it—to effectively resignify dominant health-seeking discourses, practices, and aesthetics that are experienced as injurious, including some that are particularly prevalent on Instagram. The practice of negotiating or contesting healthist discourses is not new in and of itself. For example, Phillipov (2013) demonstrates how cooking shows counter health-promotion discourses not by actively contesting them but by engaging with food on a register that renders it irrelevant, not unlike the “foodie” culture that celebrates luxury and enjoyment (Johnston and Baumann 2010). Other work has shown how lay people often negotiate with health imperatives, interpreting extreme versions of health-seeking as socially undesirable (Backett 1992; Backett et al. 1994). However, in the orthorexia community, health-seeking imperatives are spoken back to in medical terms. In other words, re-interpreting their past behaviors—their extreme diet- and fitness-oriented practices anchored in self-surveillance and self-discipline—as pathological allowed them to renounce these practices as not simply irrelevant or undesirable but as unhealthy.

The diagnosis was crucial to this process, creating an entry point for legitimately subverting widespread beliefs and practices about what constitutes healthy eating and erecting in its place an alternate paradigm. This framework signals an important rupture with conventional medical and nutritional knowledges that encourage, among other things, external regulation of food consumption, through calorie or macro-counting and weighing (think of the ubiquity of nutrition labels and recommendations) and moralized messaging about good and bad foods. By centering alternative values and bringing into question the intrinsic desirability and goodness of “healthy” foods, the recovery subculture resisted the reductionism, individualization, and decontextualization of health and illness that public health discourse is often criticized for (Arribas-Ayllon 2016; Rangel and Barry 2014; Yates-Doerr 2015).

Yet, the subversive character of the recovery subculture was not absolute. Most significantly, while disrupting assumptions about healthy eating and how to achieve it, the community ultimately retained the centrality of “health” as an organizing value. In re-signifying the meaning of pleasurable foods that are typically read as unhealthy, or in troubling interpretations of weight gain, they were not subverting health as an ideal but redefining what was understood as healthy. And unlike the “social media sad girls” analyzed by Thelandersson (2018), who refused biomedical strategies to restore them to “healthy” subjects, participants in this community aspired to recovery (i.e. true “health”) as an end goal, partially reinscribing the logics of healthism. In other words, while the content of “health” as a construct was altered, its supremacy as a “supervalue” (Crawford 1980) persisted within the recovery space. Furthermore, recovery discourse was anchored in an individualistic ethos of self-improvement and self-care—the enactment of an “entrepreneurial self” highly compatible with mainstream Instagram discourses and encouraged by the person-centric architecture of the platform (Monaghan et al. 2010; Petersen and Lupton 2000)—more focused on addressing the problem at an individual level than creating social change, even as it argued for shifting some of the blame on the culture.

The uptake of Instagram by the recovery community was also shaped by the platform’s logics, which afforded certain uses and encouraged particular practices. For example, while subcultures more generally tend to embrace their own hierarchies of what is authentic and legitimate (Thornton 1995), status in the recovery subculture was shaped by Instagram’s “economy of visibility,” which rewards being accessible to a large audience (Banet-Weiser 2018, 10) and foregrounds platform metrics as symbols of status. Central to this economy is what van Dijck (2013) calls the “popularity principle,” whereby “the more contacts you have and make, the more valuable you become” (13). Trying to build a recovery community on these terms meant that certain aesthetics and recovery performances were rewarded (whether by algorithms or the preferences of its participants) above others.

On the one hand, this incentivized very specific expressions of recovery. For example, given the popularity of transformation photos, participants who were willing to post these kinds of images could reap real benefits, but this required having experienced such physical transformations during recovery and also a willingness to circulate—and to a degree commodify—their own bodies as a means to achieve greater visibility and status, something not all were comfortable with. As Banet-Weiser (2018) notes in her analysis of popular feminism, “all bodies are not commodifiable in the same way” (25). The popularity of Ella’s transformation photos discussed above derived from both the ways in which they subverted normative health in some ways, while in others adhering to a relatively hegemonic version of white femininity. Her account’s status (i.e. following) was something she could capitalize on individually, for instance, by using it to promote recovery services or via paid sponsorships. On the other hand, this contributed to an unequal distribution of attention, and in turn, social support. Those who were unwilling or unable to use these logics to their advantage were thus less able to access external validation, which was contingent on being visible to the rest of the community.

As a platform that embeds other power structures, Instagram also contributed to reproducing other forms of privilege and exclusion within the recovery community. Language was an obvious barrier for non-English speakers (both in terms of communicating with others and attracting new followers), as was the lack of access to specific food items that were symbolically important to the subculture and which were often unavailable outside the US or Europe, or too expensive for some. The community was also largely made up of white, female (and apparently affluent) participants, which likely made the community less welcoming for participants of color and men.

Here, I have focused on the community arising around a single diagnosis (an eating disorder) and on a single platform (Instagram). Future research would benefit from examining and comparing how communities around other kinds of health conditions (mental health and beyond) negotiate medical knowledge both on and offline and how different platforms may shape the practices and communities that form on them.

## Conclusion

This study has contributed to a growing body of scholarship about diagnoses and medicalization, showing how deeply powerful a diagnostic label can be without requiring institutional or expert endorsements. It has also shown the powerful lure of building community around illness experiences that have not been satisfactorily addressed by the medical establishment, which resonates with cases beyond orthorexia. Indeed, a historically white and male profession, biomedicine has a long legacy of biases in detriment of women and people of color, whose bodies have many times been sites of harm. The Covid-19 pandemic has served as a more recent reminder of how some groups have been left out of the medical dialogue, creating a real need to fill this void elsewhere. However, there are also real risks to this, as idiosyncratic knowledges and advice can be outright incorrect or fail to help individuals in distress. The pandemic has made clear the real dangers of misinformation online, which can circulate more quickly and at a greater scale than previously possible, often among communities of like-minded individuals.

As diagnoses and medical knowledges more generally escape the confines of the medical establishment, it is pressing to understand how they are taken up and used beyond clinical settings. This requires acknowledging online spaces as very real and important sites for the formation of medical or diagnostic communities and for medical claims-making more generally. As this study has shown, these practices are both enabled and constrained by the media technologies where they come together. While digital ethnography poses real challenges, both methodologically and ethically, such methods are imperative for understanding communities like the one studied here and how they are shaped by their media environment.

## Footnotes

1 The orthorexia community also deviates from subcultures in a more traditional sense. The largely digital existence of this community differs from many of the groups examined by early subcultural literature, who came together in person. This also raises the question of the extent to which participants’ subcultural identities are disconnected from their in-person lives, where they may enact more hegemonic ways of being. That said, as Lingel (2017) shows in her scholarship on digital countercultures, subcultural communities can also congregate meaningfully online. Moreover, individuals who occupy positions of alterity in some aspects of their lives may nonetheless enact hegemonic power in others, whether on or offline.

2 While all accounts were public in the practical sense (i.e., visible to the general public), the degree to which they were open about their identities varied, ranging from complete anonymity to full disclosure.

3 Participants often used other hashtags as well, and there was significant overlap between the orthorexia recovery community and the broader eating disorder recovery (using hashtags like #edrecovery or #edwarrior), in addition to hashtags alluding to other specific diagnoses like anorexia and bulimia.

4 Quotes from users I interviewed are included under the same pseudonyms used for the interviews, and whose characteristics are available in Table 1. Quotes from users who were not interviewed are referred to more vaguely, without a name. Quotes taken from fieldnotes, rather than interviews, are labelled as such.

5 This reality points to the real limits of Instagram as a space to cultivate community (for more on how digital technologies can both help and hurt efforts to build community among subcultures, see Lingel 2017). While “community” was a recurring word participants used to describe the shared conversations and fellowship surrounding orthorexia and ED recovery on Instagram, it was not equally experienced as such by all. Some developed meaningful and reciprocal friendships, some of which translated offline to coffee dates or exchanges of phone numbers and even care-packages, but others proved unwilling or unable to forge such connections. For these users, it functioned more as an “imagined community” (Anderson 1991), effective at providing a sense of belonging and identity, but less so when it came to sociability or support (Wellman 2005).

6 The #transformationtuesday hashtag on Instagram contains over 16 million posts, most of which celebrate the abovementioned changes.
